# Targeting Bruton Tyrosine Kinase With Zanubrutinib for Treatment of Vitreoretinal Lymphoma: Report of 3 Cases

**DOI:** 10.3389/fonc.2021.676792

**Published:** 2021-04-23

**Authors:** Liang Wang, Wenxue Guan, Xiaoyan Peng

**Affiliations:** ^1^ Department of Hematology, Beijing Tongren Hospital, Capital Medical University, Beijing, China; ^2^ Beijing Institute of Ophthalmology, Beijing Tongren Eye Center, Beijing Tongren Hospital, Capital Medical University, Beijing, China

**Keywords:** vitreoretinal lymphoma, bruton tyrosine kinase, Zanubrutinib, targeted therapy, primary central nervous system lymphoma

## Abstract

Vitreoretinal lymphoma (VRL) is a rare intraocular malignancy, and standard treatment approaches have not been defined yet. Bruton tyrosine kinase inhibitors are found to be effective in the treatment of primary central nervous system diffuse large B cell lymphoma. Herein, we retrospectively reported the efficacy and safety profiles of bruton tyrosine kinase inhibitors in three consecutive patients with VRL. All three cases of VRL occurred in patients with pre-treated primary central nervous system lymphoma and the central nervous system was not involved at the time of VRL diagnosis. They were treated with zanubrutinib, a bruton tyrosine kinase inhibitor, at 160 mg twice daily orally. Rapid improvement of visual acuity and tumor control was achieved in all involved eyes of these 3 patients. Complete remission was confirmed by fundus photograph and optical coherence tomography, and the level of interleukin-10, a well-recognized biomarker for vitreoretinal lymphoma, decreased to normal in all patients. Zanubrutinib was well tolerated in all three patients, and only one adverse event of grade 3 hypertension occurred, which resolved after adjusting antihypertensive drugs. As of March 2021, these three patients have been treated with zanubrutinib for 9 months, 7 months, and 6 months, respectively, and all remained in complete remission. In conclusion, targeting bruton tyrosine kinase with zanubrutinib in vitreoretinal lymphoma is feasible and our findings can be a foundation for a paradigm shift in treatment options for this rare disease. A prospective phase 2 study evaluating the efficacy and safety of zanubrutinib in patients with vitreoretinal lymphoma is ongoing to validate our findings (ChiCTR2000037921).

## Introduction

Vitreoretinal lymphoma (VRL) is a rare intraocular malignancy where the lymphocytic neoplastic cells affect mainly in the vitreous and/or retina. It is regarded as a part of the primary central nervous system lymphoma (PCNSL) and shows a close relationship with CNS progression, with a median progression-free survival of 3.5 months in the PCNSL patients with VRL (vs. 8.3 months in those without VRL) (1, 2). Currently, no standard treatment approaches have been defined yet, although intravitreal chemotherapy using methotrexate combined with systemic chemotherapy are generally used in the treatment of VRL.

Bruton tyrosine kinase (BTK) is a vital effector molecule in the progress of B-cell proliferation. VRL, as well as PCNSL, display typically an activated B cell-like (ABC) phenotype of diffuse large B-cell lymphoma (DLBCL), with frequent CD79B and MYD88 mutations (3, 4), which may represent a strong biological rationale for the use of BTK inhibitors in the treatment of PCNSL and VRL. Previous studies have demonstrated that BTK inhibitors could penetrate the blood-brain barrier, and achieve 70–90% response rate in patients with PCNSL (3–5). However, whether BTK inhibitors could penetrate the blood-eye barrier and provide benefits to patients with VRL remains unknown. Herein, we reported our experience with zanubrutinib, a novel BTK inhibitor, in three consecutive patients with VRL. All three cases of VRL occurred in patients with pre-treated primary central nervous system lymphoma and the central nervous system was not involved at the time of VRL diagnosis. The treatment results could provide rationality for our ongoing prospective phase 2 study (ChiCTR2000037921).

## Methods

Three consecutive patients diagnosed as VRL in the Eye Center of Beijing Tongren Hospital were enrolled in this study. Vitreous biopsy was done in all patients to confirm the diagnosis of VRL. All the three patients had pre-treated PCNSL and the CNS was not involved at the time of VRL diagnosis, which was confirmed by magnetic resonance imaging (MRI) of brain and cerebrospinal fluid (CSF) examination. In addition, cytokine analysis (including IL-10 and IL-6) in the anterior chamber of the eye was done using Cytometric Bead Array (CBA). Following written consent and after exclusion of contraindications, all patients were treated monotherapy with oral zanubrutinib 160 mg twice daily continuously, until disease progression or unaccepted toxicities. Detailed clinical features, treatment outcomes and adverse events were recorded. This study was approved by the IRB of Beijing Tongren Hospital.

## Results

### Case 1

**Figure 1:**
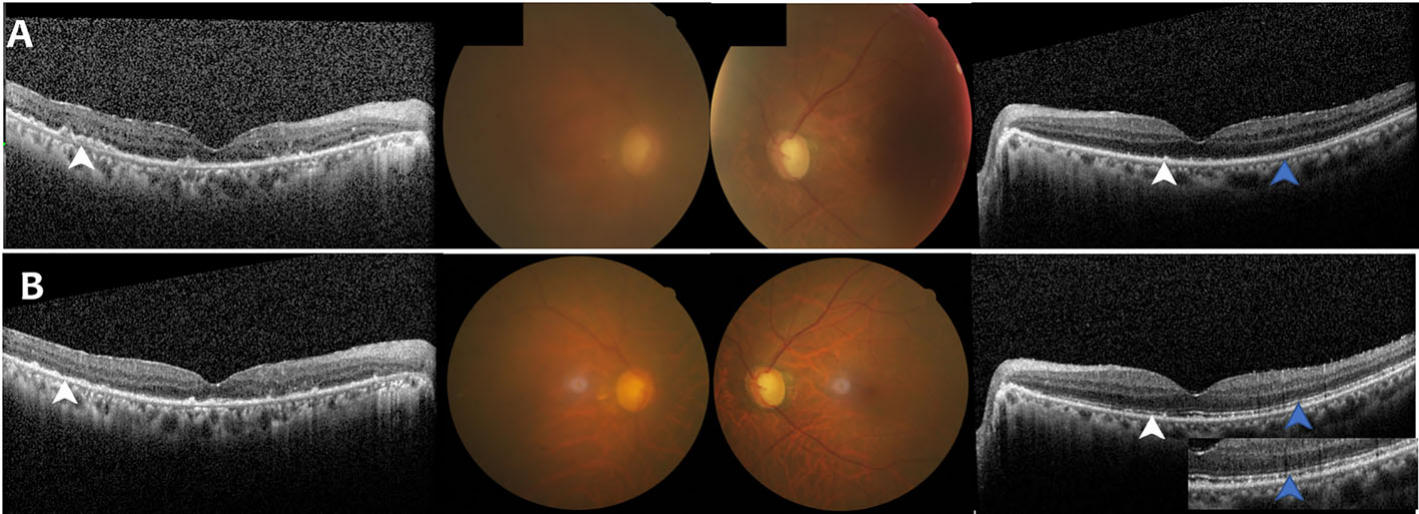
Case 1: A 66-Year-Old Man with Bilateral Vitreoretinal Lymphoma. **(A)** Fundus photograph and optical coherence tomography (OCT) findings at initial presentation showed binocular vitreous opacities and disorganization of outer retinal architecture (blue arrow) with numerous subretinal or subretinal pigment epithelial (RPE) hyperreflective infiltration (white arrow). **(B)** Fundus photograph and OCT findings after Zanubrutinib treatment demonstrated binocular remission of the vitreous opacities and reduced number of subretinal or sub-RPE lesions (white arrow) with partial restoration of the structure in outer retina (blue arrow).

A 66-year-old man with pretreated PCNSL (DLBCL) developed bilateral VRL, which was confirmed by vitreous biopsy. He was previously treated with six cycles of rituximab and high-dose methotrexate (HD-MTX), followed by lenalidomide maintenance for one year, until symptoms developed in the eyes. He demonstrated severe vitreous opacities in the right eye and mild opacities in the left eye, with binocular disorganization of outer retinal architecture and numerous subretinal or subretinal pigment epithelial (RPE) hyperreflective infiltration (**Figure 1A**). Visual acuity was 20/100 OD and 20/50 OS. Cytokine analysis showed interleukin (IL)-10 levels increased to 1104.9pg/ml and 20.9pg/ml (normal range: 0-5.0pg/ml) and IL-10/IL-6 ratio was 4.2 and 9.5 in the right and left eye, respectively (positive value>1). Both positron emission tomography-computed tomography (PET-CT) scan and MRI scan revealed no residual lesions in the brain and the examination of CSF using flow cytometry was normal. He was treated with oral zanubrutinib 160 mg twice daily. Three days later, he perceived a significant improvement in vision. One month after initiation of treatment, binocular significant remission of the vitreous opacities and reduced number of subretinal or sub-RPE lesions with restoration of the structure in outer retina was noted (**Figure 1B**). Final visual acuity improved to 20/100 OD and 20/20 OS. IL-10 levels dropped to normal and IL-10/IL-6 ratio was less than 1 in both eyes. This patient was still on treatment with zanubrutinib and remained complete remission after nine months of follow up. Grade 3 hypertension occurred and the dosage of anti-hypertension drug was doubled to control the blood pressure. No bone marrow suppression, bleeding, diarrhea, arthritis, and atrial fibrillation occurred.

### Case 2

**Figure 2:**
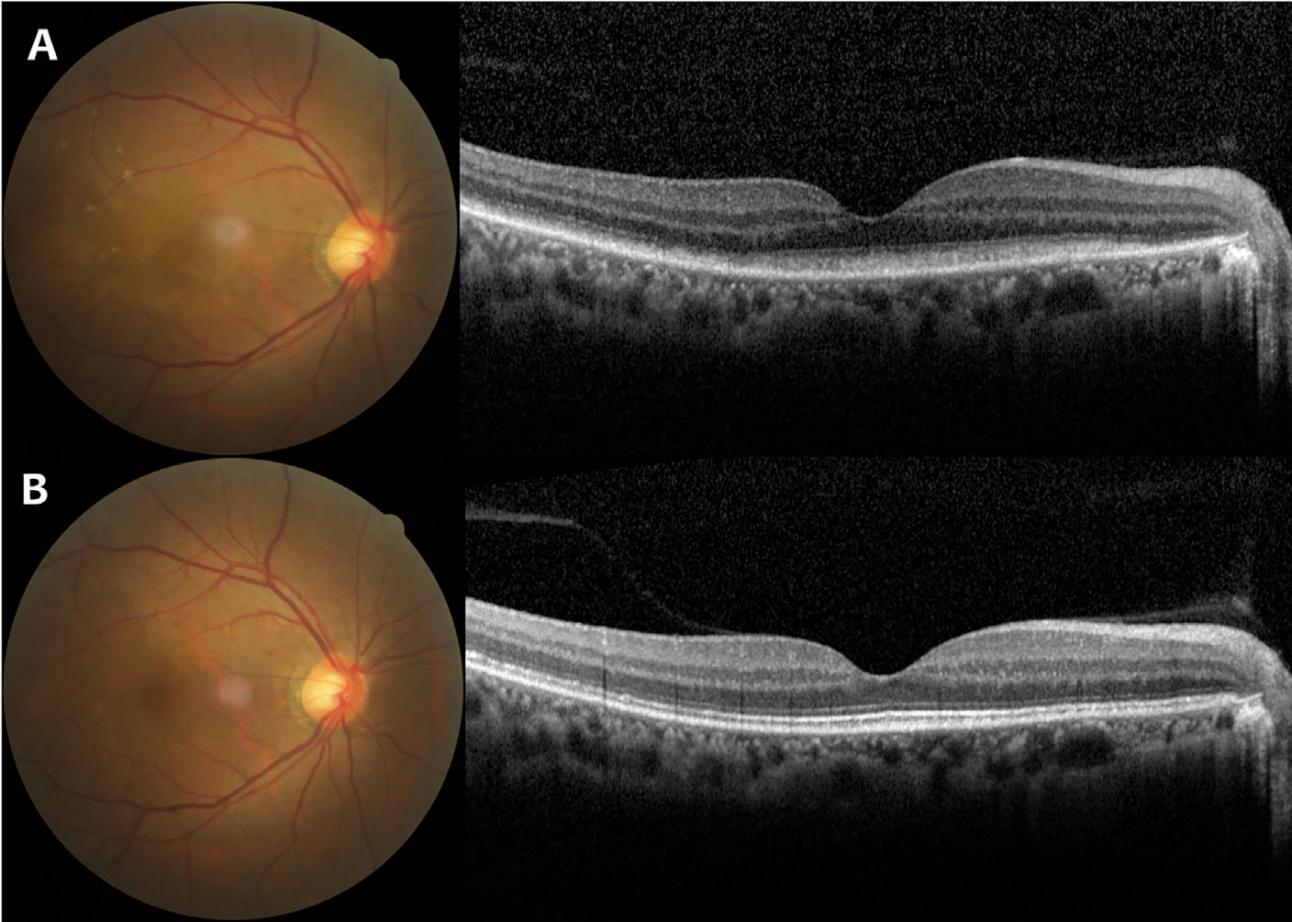
Case 2: A 41-Year-Old Woman with Vitreoretinal Lymphoma in the Right Eye. **(A)** Fundus photograph and optical coherence tomography (OCT) findings at initial presentation showed several round whitish lesions in the temporal to superior area with mild vitreous opacities and subretinal infiltration. **(B)** Fundus photograph and OCT findings after Zanubrutinib treatment demonstrated the whitish lesions subsided with complete clearance of vitreous and retinal structures.

A 41-year-old woman with pretreated PCNSL (DLBCL) developed VRL in the right eye and demonstrated tumor infiltration of the vitreous and retina (**Figure 2A**), which was also confirmed by vitreous biopsy. She previously received six cycles of rituximab and HD-MTX, and no consolidation or maintenance therapy was given after achievement of complete remission. Six months after the end of HD-MTX treatment, symptoms developed in her right eye. Visual acuity was 20/63 OD and 20/13 OS. Cytokine analysis showed IL-10 level increased to 724.3pg/ml and IL-10/IL-6 ratio was 51.0. The MRI scan and CSF examination ruled out the residual lesions in the brain. She was treated with oral zanubrutinib 160 mg twice daily continuously. One month later, the vitreous and retinal structures were completely clear and visual acuity was improved to 20/25 with a significant reduction in IL-10 level in the right eye, (**Figure 2B**). The left eye remained normal. This patient was still in remission after seven months of follow up, and no adverse events occurred.

### Case 3

**Figure 3:**
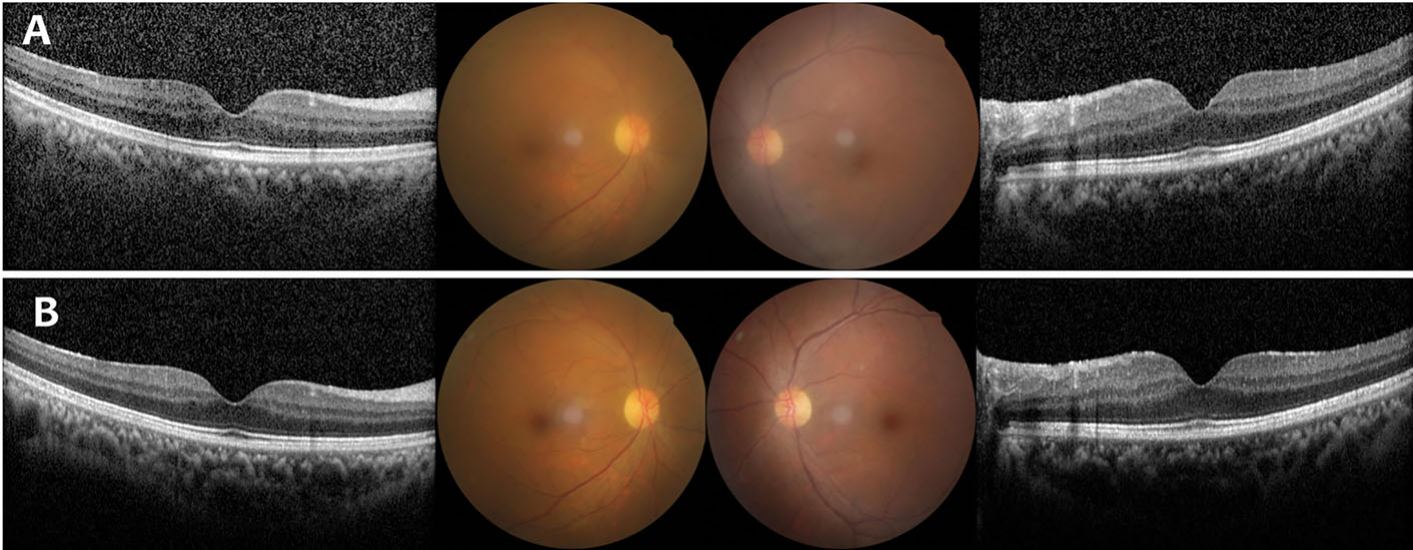
Case 3: A 50-Year-Old Woman with Bilateral Vitreoretinal Lymphoma. **(A)** Fundus photograph and optical coherence tomography (OCT) findings at initial presentation showed binocular severe vitreous opacities without retinal infiltration. **(B)** Fundus photograph and OCT findings after Zanubrutinib treatment demonstrated complete vitreous tumor clearance in both eyes.

A 50-year-old woman with biopsy confirmed PCNSL (DLBCL) developed bilateral VRL after complete remission of PCNSL. She complained of shadows fluttering before her eyes. Clinical examination demonstrated binocular severe vitreous opacities without retinal infiltration (**Figure 3A**). Visual acuity was 20/50 OD and 20/32 OS. Cytokine analysis showed IL-10 level increased to 135.9pg/ml and 218.9pg/ml with binocular IL-10/IL-6 ratio greater than 1. She was treated with oral zanubrutinib 160 mg twice daily continuously. The symptom of floating shadows resolved completely within a week. One month after treatment, the IL-10 level dropped to normal with complete vitreous tumor clearance in both eyes (**Figure 3B**). Zanubrutinib was well tolerated by this patient without any adverse events being reported. Complete remission was maintained after six months of follow up.

## Discussion

VRL typically occurs in older individuals with a median age of 60 years, usually masquerading as chronic posterior uveitis and presenting with vitreous haze and retinal/subretinal infiltrations. VRL is supposed to be highly suspected when a patient with PCNSL presents with the clinical manifestations described above. Moreover, the IL-10 level and IL-10/IL-6 ratio of aqueous fluid and OCT imaging are helpful diagnostic tools and can be used to evaluate treatment outcomes (6, 7).

Intravitreal methotrexate, as the most common treatment option for VRL, has been shown to effectively control tumor with a total of 25 injections. Intraocular chemotherapy avoids the serious systemic side effects, but it commonly leads to ocular toxicity including corneal epitheliopathy, cataract and ocular hypertension (1). Moreover, it is inconvenient for both patients and physicians, because intraocular chemotherapy has to be done in the ward, and many patients were unable to be admitted inpatient during the pandemic of COVID-19. Thus, a more convenient outpatient treatment was urgently desired for patients with VRL. Furthermore, it is controversial whether systemic chemotherapy should be initiated again in patients with PCNSL that recurs only in the eyes due to severe systemic side effects.

A new molecular subtype of DLBCL, the so-called MCD subtype, characterized by the co-occurrence of MYD88 and CD79B gene mutations has been proposed, with an inferior outcome (8). It has been reported that nearly half of patients with VRL or PCNSL could be classified as MCD subtype (3, 4), and the BTK inhibitor ibrutinib results in an overall response rate of 80% in patients with MCD subtype of DLBCL (9). A study by C. Soussain et al. reported a 100% disease control rate in 14 PCNSL patients with intraocular involvement using ibrutinib treatment for 2 months (3), however, detailed information about relief of ocular symptoms and change of cytokine levels were not provided. In this study, all the three patients had pre-treated PCNSL and the CNS was not involved at the time of VRL diagnosis. Zanubrutinib can result in complete remission of intraocular lymphoma within 1 month with no evidence of recurrence in the CNS, which means that it can permeate both the blood-eye barrier and blood-brain barrier to control intraocular lymphoma and may have a preventive effect on the CNS. The level of IL-10 and ratio of IL-10/IL-6 were found to be highly sensitive and specific to VRL (7), and regular monitoring of those cytokine levels could be useful in response assessment and early detection of disease recurrence. In this study, all patients had rapid normalization of IL-10 level after one month of zanubrutinib treatment, and remained undetectable after follow up of 6-9 months, indicating durable response achieved by BTK inhibitors. Zanubrutinib was well tolerated in our study, and did not reveal any new adverse events. The grade 3 hypertension occurred in the first patient was well controlled by supportive care.

However, there are several limitations in our study. First, the concentration of zanubrutinib in the anterior chamber of the eye and vitreum was not tested, and these results could provide direct evidence concerning the ability of penetration of zanubrutinib across the blood-eye barrier. Second, the number of patients reported here are too small to draw any robust conclusions. Thus, we are conducting a prospective phase 2 study to evaluate the efficacy and safety profiles of BTK inhibitors monotherapy for VRL in order to significantly improve the prognosis and quality of life of these patients (Registration number: ChiCTR2000037921). Moreover, serial samples of aqueous fluid and peripheral blood will be collected to detect the concentrations of BTK inhibitors. The results of this study will provide a robust foundation for a paradigm shift in the treatment of this rare disease.

In conclusion, this series of 3 cases showed feasibility and short term efficacy of zanubrutinib in patients with VRL. A much larger study with longer follow up duration is needed to substantiate the results observed by us.

## Data Availability Statement

The original contributions presented in the study are included in the article/supplementary material. Further inquiries can be directed to the corresponding author.

## Ethics Statement

The studies involving human participants were reviewed and approved by Institutional Review Board of Beijing TongRen Hospital. The patients/participants provided their written informed consent to participate in this study.

## Author Contributions 

XP and LW conceived and designed the study. WG collected the clinical data. WG and LW wrote the paper. All authors contributed to the article and approved the submitted version.

## Funding

This work was financially supported through grants from Beijing Advanced Innovation Center for Big Data-Based Precision Medicine, Beijing Tongren Hospital, Beihang University & Capital Medical University (grant No. BHTR-KFJJ-202009) to LW and Key Research Program of Beijing Institute of Ophthalmology (No: 2019005) to XP.

## Conflict of Interest

The authors declare that the research was conducted in the absence of any commercial or financial relationships that could be construed as a potential conflict of interest.
